# Angiomotin-p130 inhibits β-catenin stability by competing with Axin for binding to tankyrase in breast cancer

**DOI:** 10.1038/s41419-019-1427-2

**Published:** 2019-02-21

**Authors:** Jiao Yang, Xiaoman Zhang, Zheling Chen, Yanwei Shen, Fan Wang, Yaochun Wang, Yu Liu, Peijun Liu, Jin Yang

**Affiliations:** 1 Department of Medical Oncology, The First Affiliated Hospital of Xi’an Jiaotong University, Xi’an, China; 2 Center for Translational Medicine, The First Affiliated Hospital of Xi’an Jiaotong University College of Medicine, Xi’an, China; 3 Department of Biology and Biochemistry, University of Houston, Houston, TX USA

## Abstract

Growing evidence indicates that Angiomotin (Amot)-p130 and Amot-p80 have different physiological functions. We hypothesized that Amot-p130 is a tumor suppressor gene in breast cancer, in contrast with the canonical oncogenicity of Amot-p80 or total Amot. To clarify the role of Amot-p130 in breast cancer, we performed real-time quantitative PCR, western blotting, flow cytometry, microarray, immunofluorescence, immunoprecipitation, and tumor sphere-formation assays in vitro, as well as tumorigenesis and limited-dilution analysis in vivo. In this study, we showed that Amot-p130 inhibited the proliferation, migration, and invasion of breast cancer cells. Interestingly, transcriptional profiles indicated that genes differentially expressed in response to Amot-p130 knockdown were mostly related to β-catenin signaling in MCF7 cells. More importantly, most of the downstream partners of β-catenin were associated with stemness. In a further validation, Amot-p130 inhibited the cancer stem cell potential of breast cancer cells both in vitro and in vivo. Mechanistically, Amot-p130 decreased β-catenin stability by competing with Axin for binding to tankyrase, leading to a further inhibition of the WNT pathway. In conclusions, Amot-p130 functions as a tumor suppressor gene in breast cancer, disrupting β-catenin stability by competing with Axin for binding to tankyrase. Amot-p130 was identified as a potential target for WNT pathway-targeted therapies in breast cancer.

## Introduction

Breast cancer (BCa) is the most common cancer in the female population, showing the highest incidence and prevalence among female cancers^1^. Although precision therapy has improved BCa survival, most patients inevitably suffer from disease recurrence or metastasis. It is, therefore, important to explore the potential mechanism underlying breast carcinogenesis.

Angiomotin (Amot) was initially discovered as an angiostatin-binding protein that regulates endothelial cell migration and tube formation^2^. Amot has two classic isoforms, Amot-p130 and Amot-p80. They are nearly identical except that Amot-p130 has an N-terminal glutamine-rich domain containing one LPTY and two PPXY sequences. This extended domain mediates many protein–protein interactions. Recent studies have reported conflicting data regarding the role of Amot in different cancers^3–6^. Amot has been shown to play both oncogenic and tumor suppressive roles even in the same cancer type (BCa and hepatic cancer)^6–9^.

Amot is expressed at higher levels in BCa tissues than in para-carcinoma tissues and promotes the proliferation and invasion of BCa cells through the YAP/TAZ pathway^10^. Amot-p80 promotes proliferation and invasion in BCa cells^11^, and DNA vaccines targeting Amot-p80 inhibit tumor growth and metastasis in vivo^12,13^. However, Amot-p130 has been shown to inhibit the proliferation of non-cancerous breast epithelial cells^14^.

Amot isoforms have distinct physiological functions. During embryonic development, Amot-p80 is expressed early, whereas Amot-p130 is expressed later^15^. In endothelial cells, Amot-p80 is found at the leading edge of migrating cells and diffuses throughout the cytoplasm when not migrating, whereas Amot-p130 is primarily located at cell junctions^16^. The difference between the isoforms is also apparent in the regulation of endothelial cell migration, in which Amot-p80 and Amot-p130 play promotive and inhibitive roles, respectively^17–19^. The Amot-p80/Amot-p130 ratio is used as an indicator of migration activity^20,21^. We hypothesized that Amot-p80 and Amot-p130 have different functions in breast carcinogenesis. In a previous work from our group, we have shown that Amot-p130 decreases the motility of BCa cells^22^. Here, we have investigated the link between the inhibition of metastasis and Amot-p130 in BCa.

Amot-p130 shows a high structural homology with AmotL2^23^. AmotL2 inhibits WNT signaling by trapping β-catenin in recycling endosomes^24^. However, it is unclear whether Amot-p130 regulates the WNT/β-catenin pathway. In the present study, the modulation of Amot-p130 expression revealed that Amot-p130 inhibited the cancer stem cell (CSC) potential of BCa, disrupting β-catenin stability by competing with Axin for binding to tankyrase (TNKS), leading to a further inhibition of cell proliferation and epithelial–mesenchymal transition (EMT) in BCa.

## Results

### Amot-p130 inhibits the proliferation of BCa cells

**Fig. 1:**
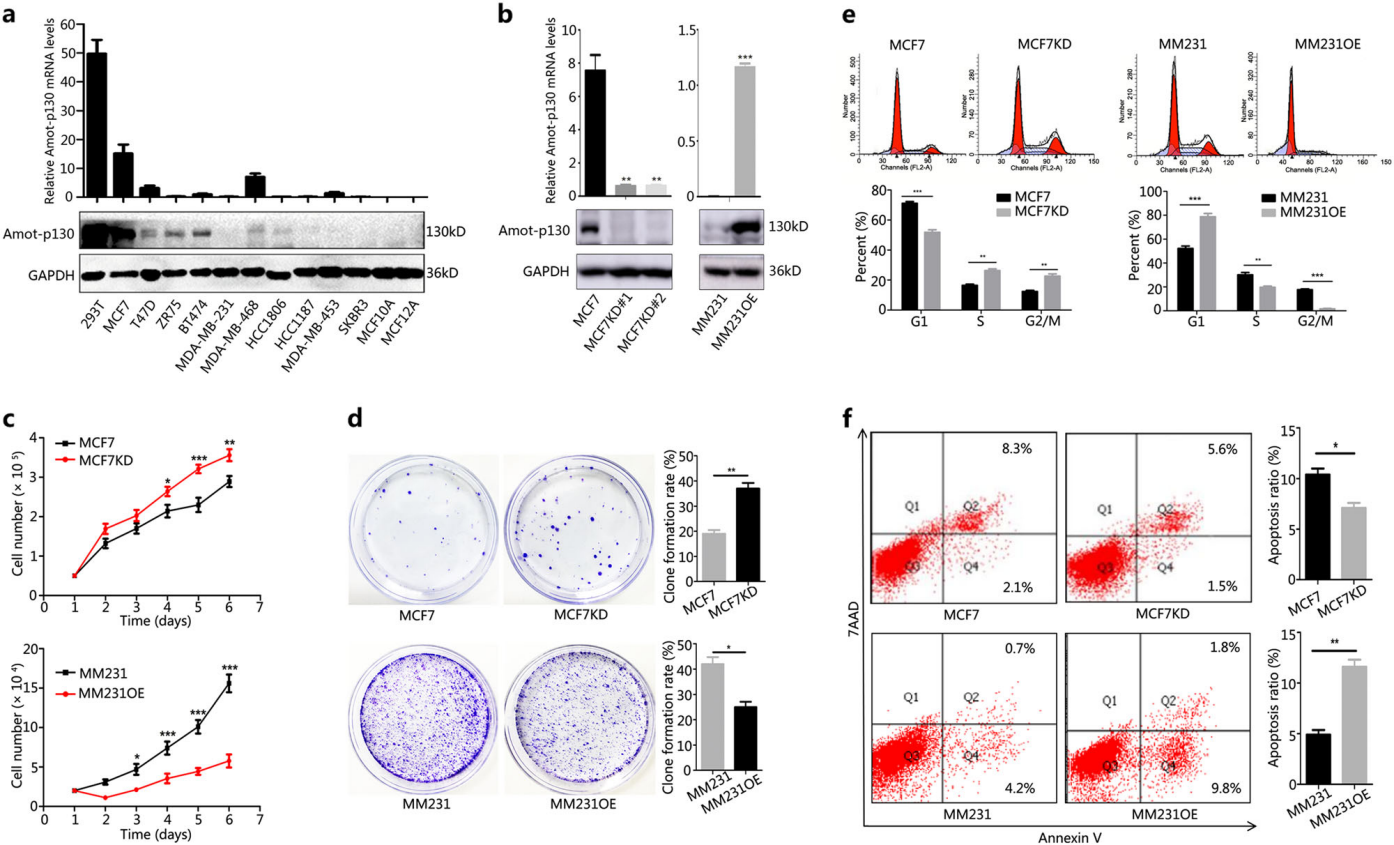
Amot-p130 inhibits the proliferation of breast cancer cells. **a** Expression levels of Amot-p130 in nine breast cancer cell lines and two immortalized breast epithelial cells (MCF10A and MCF12A) (using 293T cells as the positive control) as determined by (upper) RT-qPCR and (lower) western blotting. GAPDH was used as the loading control. **b** Interference efficiency of Amot-p130 was evaluated by (upper) RT-qPCR and (lower) western blotting. GAPDH was used as the loading control. **c** Cell proliferation was measured using cell count assay. **d** Cell proliferation was measured using plate clone formation. Clones were stained with 0.4% crystal violet (left). Numbers of clones are shown as the mean ± SD of three independent experiments (right). **e** Cell cycles were determined by flow cytometry (upper). The proportion of cells distributed in each cell cycle was calculated from three independent experiments (lower). **f** Cell apoptosis was determined by flow cytometry (left). Numbers of apoptotic cells were calculated from three independent experiments (right). MM231 is the abbreviation of MDA-MB-231. All *P* values were calculated by paired Student’s *t* test. *P < 0.05, **P < 0.01, ***P < 0.001. RT-qPCR, real-time quantitative PCR

The basal expression of Amot-p130 varied significantly among the different BCa cell lines (Fig. 1a), showing lower expression levels in basal-like cell lines than in luminal cell lines. MCF7 with Amot-p130 knockdown (MCF7KD) and MM231 with Amot-p130 overexpression (MM231OE) cells were established using Amot-p130-targeted lentivirus (Fig. 1b) to determine the role of Amot-p130 in cell proliferation. The results of the cell count assay showed that MCF7KD cells grew faster, whereas MM231OE cells grew at a slower rate than control cells (Fig. 1c). Consistently, the extent of colony formation was higher in MCF7KD (42% vs 25%) and lower in MM231OE cells than in control cells (19% vs 37%) (Fig. 1d). An increase in the percentage of MCF7KD cells in S and G2/M phases occurred concomitantly with a decrease in MM231OE S- and G2/M-phase cells (Fig. 1e). Apoptosis was consistently decreased in MCF7KD cells (10.4% vs 7.1%) and increased in MM231OE cells (4.9% vs 11.6%) (Fig. 1f).

### Amot-p130 inhibits the EMT of BCa cells

**Fig. 2:**
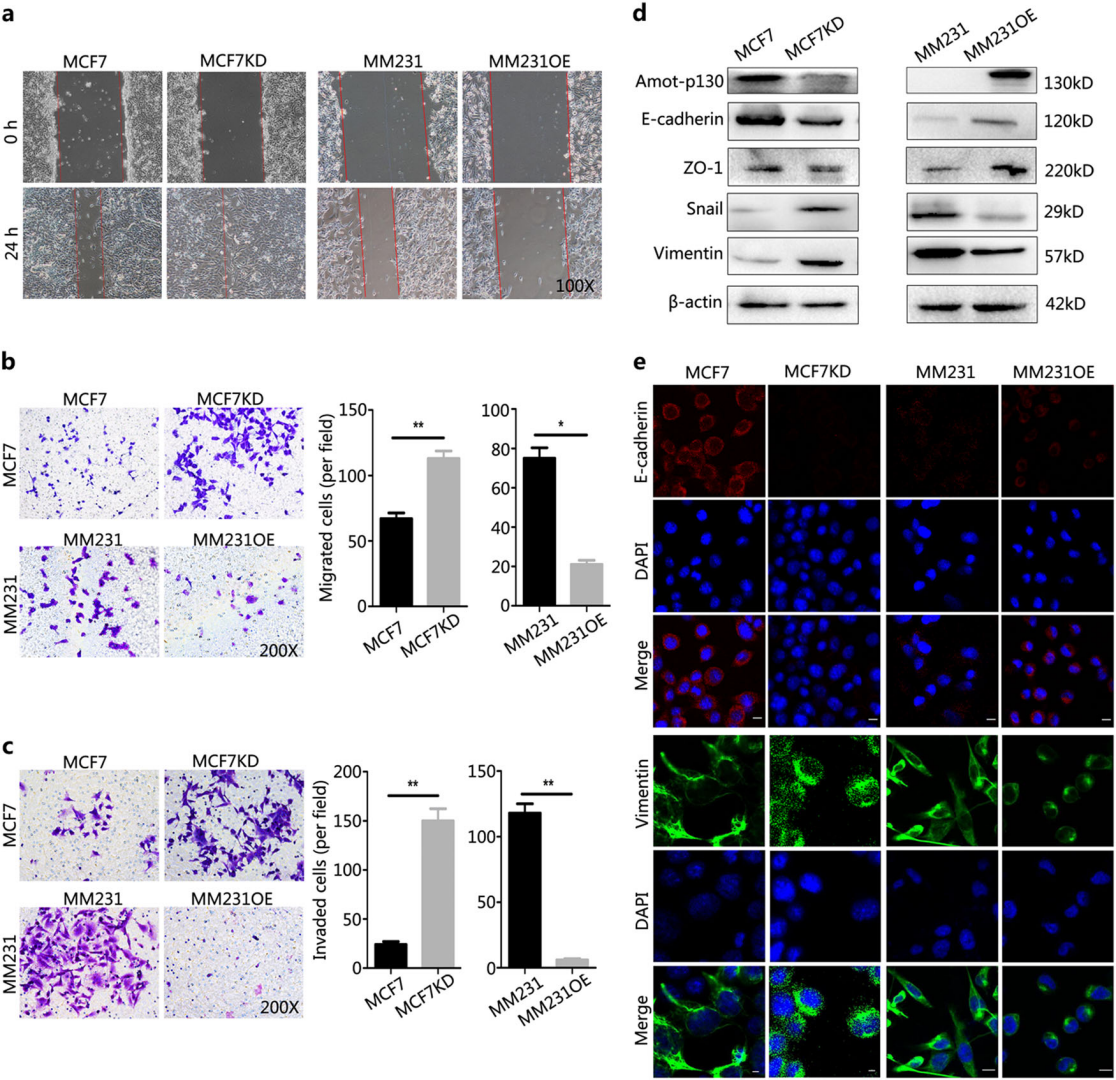
Amot-p130 inhibits the EMT of breast cancer cells. **a** A photomicrograph of a scratch created with a 200 μl pipette tip in the wound-healing assay at 0 and 24 h. **b** Cell migration and **c** cell invasion were assessed using transwell assay (left). The number of migrated or invaded cells was expressed as the mean ± SD of three independent experiments (right). **d** Expression of epithelial to mesenchymal transition (EMT) markers was determined by western blotting. β-actin was the loading control. **e** The subcellular localization of EMT markers was determined by immunofluorescence staining. Both E-cadherin and vimentin antibodies were diluted at a ratio of 1:200. DAPI was used for nuclear staining (blue). Scale bars = 25 µm. All *P* values were calculated by Student’s *t* test. *P < 0.05, **P < 0.01. DAPI, 4′,6-diamidino-2-phenylin

The activation of EMT allows cancer cells to acquire migratory, invasive, and CSC potential. In wound-healing assays, the recovery of confluency was faster in MCF7KD cells and markedly slower in MM231OE cells than in control cells at 24 h post-scratch (Fig. 2a). The number of cells migrating across the Matrigel was higher in MCF7KD (113) than in MCF7 control cells (67), whereas that in MM231OE cells was less than one-third of those in the MM231 controls (21 vs 75) (Fig. 2b). The results of invasion assays showed that the number of invading cells was more than six folds higher in MCF7KD than in MCF7 cells (24 vs 150) and less than one-tenth in MM231OE than in MM231 cells (118 vs 6) (Fig. 2c). The EMT phenotype of cancer cells is marked by the balance between mesenchymal and epithelial markers. In MCF7KD cells, mesenchymal markers, including vimentin and Snail, were upregulated, whereas epithelial markers, including E-cadherin and ZO-1, were downregulated compared with those in MCF7 cells (Fig. 2d). By contrast, mesenchymal marker expression was lower, whereas epithelial marker expression was higher in MM231OE than in MM231 cells. Specifically, E-cadherin disappeared from the membrane and vimentin was transferred from the cytoplasm to the nucleus in MCF7KD cells (Fig. 2e). Consistently, E-cadherin appeared in the membrane and vimentin transitioned from a diffuse pattern to a dense dot in the cytoplasm of MM231OE cells. Collectively, these results suggested that Amot-p130 inhibits proliferation and EMT in BCa cells.

### Identification of β-catenin as an effector of Amot-p130 in BCa

**Fig. 3:**
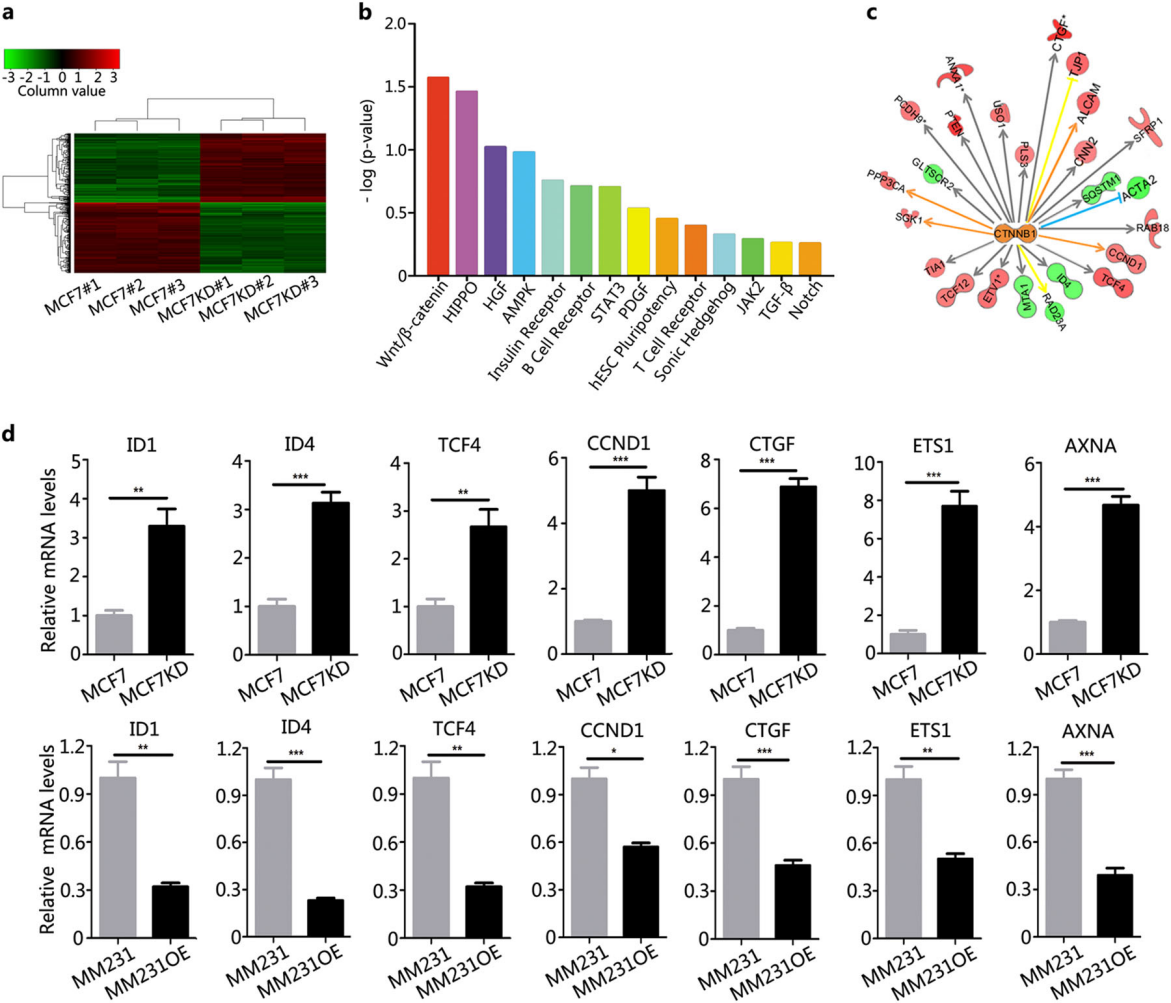
Identification of β-catenin as an effector of Amot-p130 in breast cancer cells. **a** A visualized heatmap of differentially expressed genes in MCF7KD cells compared to MCF7 control cells. Red color indicates upregulation and green indicates downregulation (detailed data are uploaded as Supplementary Table S1). **b** Functionally associated pathways were identified by Ingenuity Pathway Analysis. Enriched pathways related to breast carcinogenesis were sorted by Fisher's exact *P* values. **c** An interactive network analysis of the key molecular β-catenin from the first ranked WNT pathway. **d** Expression validation of the genes from the interactive network by quantitative PCR. The means ± SD of relative fold changes from triplicate experiments were plotted. GAPDH was used as the control. *P* values were calculated by paired Student’s *t* test

The molecular mechanisms underlying the function of Amot-p130 in BCa were examined using transcriptome microassays. A total of 255 genes were upregulated and 251 genes were downregulated in MCF7KD cells compared with MCF7 cells (Fig. 3a, Supplementary Table S1). The annotations of these differentially expressed genes were significantly related to Wnt/β-catenin signaling in breast carcinogenesis (Fig. 3b). β-Catenin was identified as the pivotal link among all deregulated genes (Fig. 3c). All β-catenin downstream partners, including *ID1, ID4, TCF4, CCND1, CTGF, ETS1*, and *AXNA*, were associated with CSC potential. Using real-time quantitative PCR, we confirmed that the expression of these genes was higher in MCF7KD than in MCF7 cells and lower in MM231OE than in MM231 cells (Fig. 3d). Taken together, these results indicate that Amot-p130 may affect the CSC potential of BCa by modulating β-catenin signaling.

### Amot-p130 inhibits the CSC potential of BCa

**Fig. 4:**
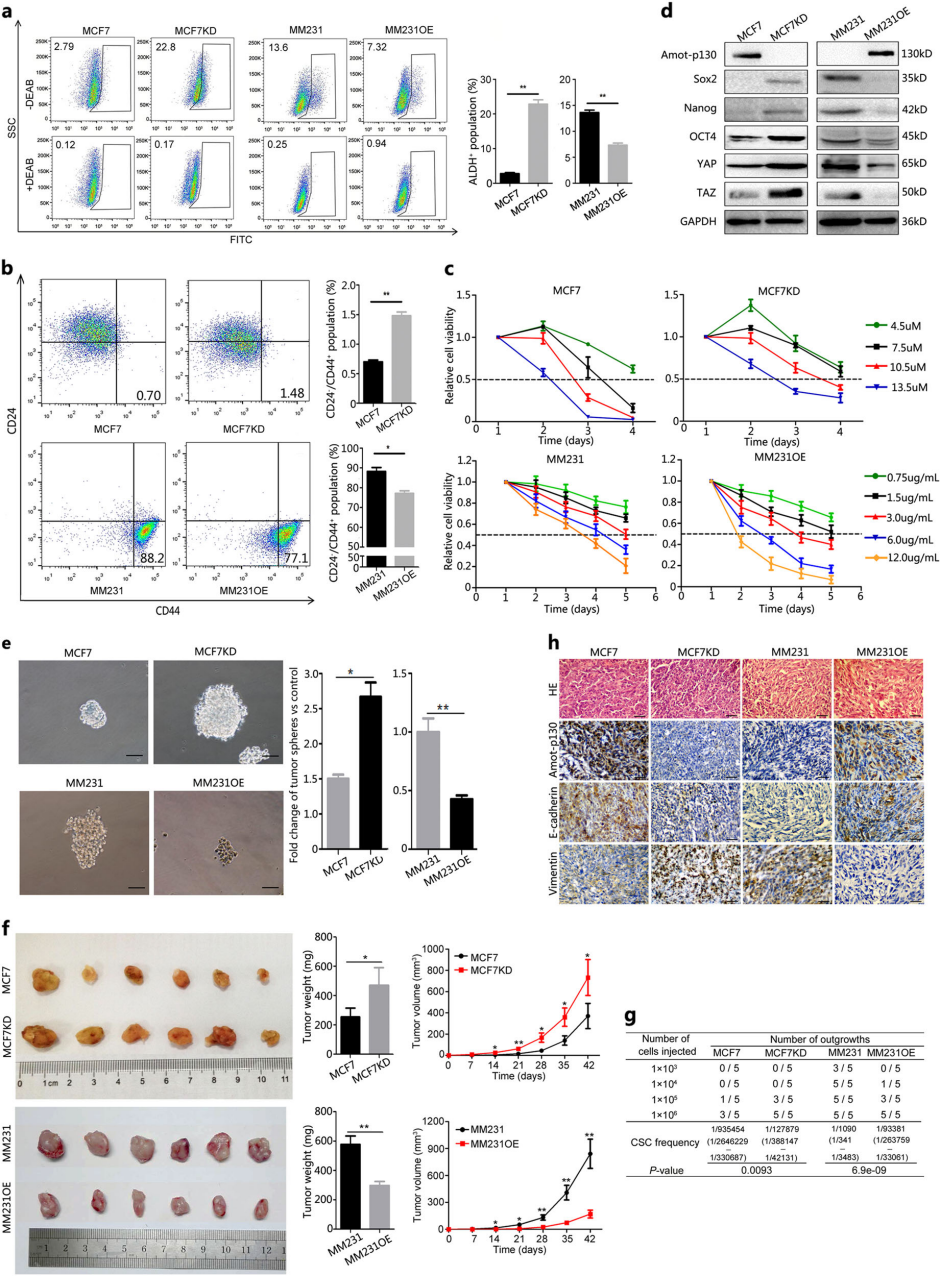
Amot-p130 inhibits the CSC potential of breast cancer cells. **a** ALDH-positive population was detected by flow cytometry (left). The proportions were calculated from three independent experiments (right). **b** CD24-low/CD44-high populations were detected by flow cytometry (left). The proportions were calculated from three independent experiments (right). **c** The tolerance of MCF7 cells to tamoxifen and MM231 cells to cisplatin was determined by MTT assay. **d** Stemness markers were assessed by western blotting. GAPDH was used as the loading control. **e** The formation of tumor spheres was recorded under microscopy (left). Scale bar = 50 µm. The proportions were calculated from three independent experiments (right). **f** Xenograft formation in NOD-SCID mice evaluated at 6 weeks after injection. Tumor volumes were determined using the formula volume = 1/2 × length × width^2^. **g** The number of outgrowths was recorded at the end of the 6th week using limited-dilution assays. **h** IHC analysis of protein expression of Amot-p130, E-cadherin, and vimentin in xenograft tissues. Scale bar = 200 µm. All *P* values were calculated by paired Student’s *t* test. *P < 0.05, **P < 0.01. IHC, immunohistochemistry; MTT, 3-(4,5-dimethylthiazol-2-yl)-2,5-diphenyltetrazolium bromide

Based on the microarray results, we assessed the effect of Amot-p130 on the CSC potential of BCa. ALDH-positive populations or CD24-low/CD44-high populations are believed to contain breast CSC subsets^25,26^. In vitro, the percentages of ALDH-positive cells increased from 2.79% to 22.8% in MCF7KD compared with MCF7 cells, whereas it decreased from 13.6% to 7.32% in MM231OE compared with MM231 cells (Fig. 4a, b). Similarly, the proportion of CD44-high/CD24-low cells was higher in MCF7KD (1.48% vs 0.70%) and lower in MM231OE (77.1% vs 88.2%) cells than in the controls. CSCs play an important role in drug resistance. Amot-p130 knockdown increased the resistance of MCF7 cells to tamoxifen, whereas Amot-p130 overexpression induced susceptibility to cisplatin in MM231 cells (Fig. 4c). In addition, the stem cell markers *Sox2, Nanog, OCT4, YAP*, and *TAZ* were upregulated in MCF7KD and downregulated in MM231OE cells (Fig. 4d). MCF7KD cells formed a two folds higher number of tumor spheres and with a larger size than the control cells, whereas MM231OE cells formed fewer (33 vs 76) and smaller spheres than control cells (Fig. 4e).

Next, we examined the ability of Amot-p130 to suppress tumorigenesis in vivo using a nude mouse xenograft model. MCF7KD cell injection induced the formation of larger tumors, whereas MM231OE cell injection induced smaller tumors than those in the respective control cells (Fig. 4f). The limited dilution-condition assay revealed that the rate of tumor formation was higher in MCF7KD and lower in MM231OE cells than in the respective controls (Fig. 4g). Immunohistochemistry (IHC) staining showed that E-cadherin was downregulated and vimentin was upregulated in MCF7KD cell-derived xenograft tissues (Fig. 4h). Conversely, MM231OE-derived xenograft tissues showed E-cadherin upregulation and vimentin downregulation. Taken together, these results indicated that Amot-p130 suppressed the CSC potential of BCa.

### Amot-p130 inhibits the WNT pathway without direct interaction with β-catenin

**Fig. 5:**
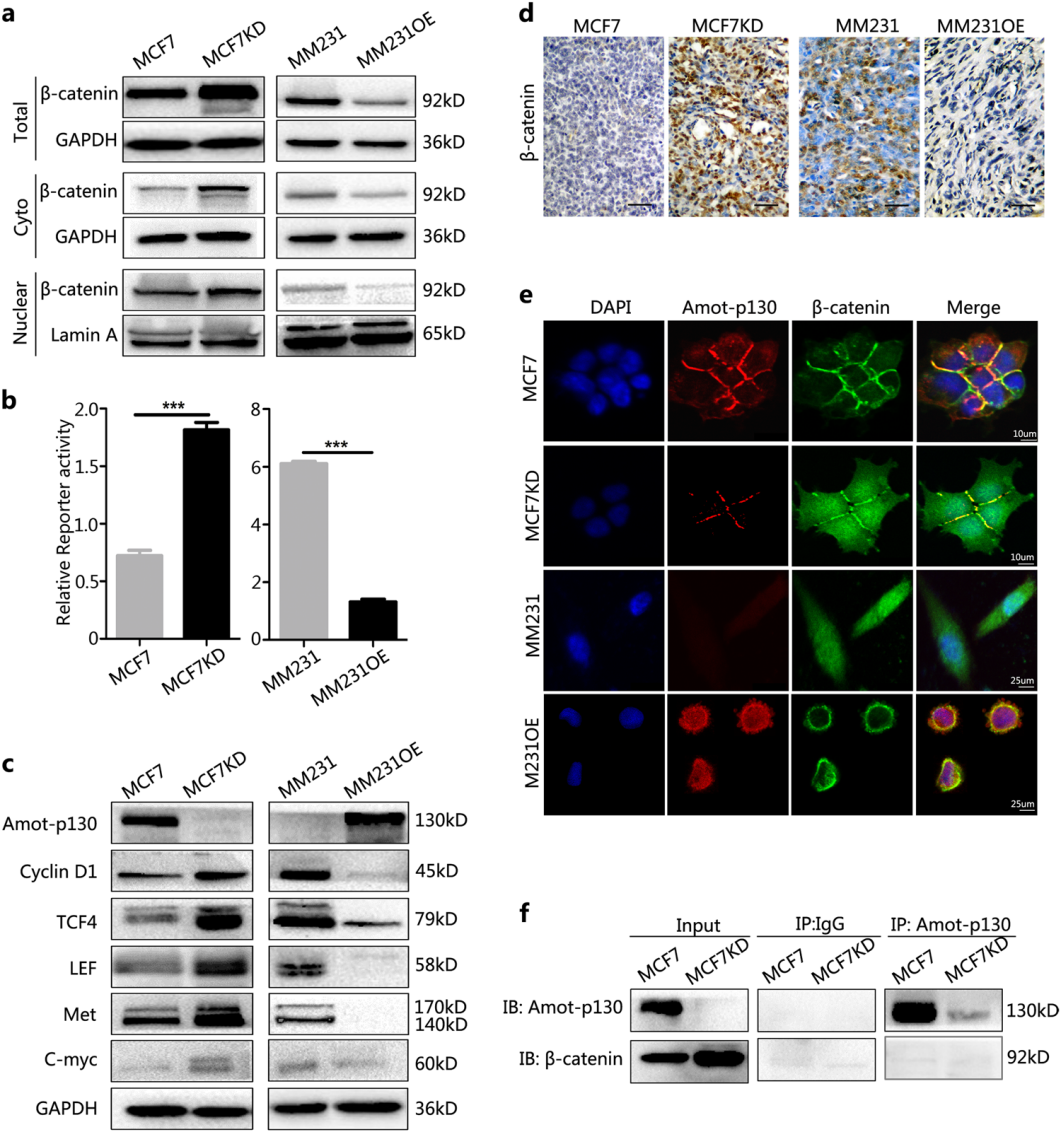
Amot-p130 inhibits WNT pathway activation in breast cancer cells. **a** Protein levels of total β-catenin, cytoplasmic β-catenin, and nuclear β-catenin as determined by western blotting. GAPDH was used as the loading control for the total and cytoplasmic protein. Lamin A was used as the loading control for nuclear protein. **b** β-Catenin-driven transcription activity was determined by TOP/FOP luciferase reporter assays. Normalization was based on internal Renilla luciferase actvity. The final reporter activity was measured as TOP/FOP ratio and was expressed as the mean ± SD of three independent experiments. **c** Protein levels of WNT downstream targets were determined by western blotting. **d** β-Catenin expression was determined by immunohistochemistry staining in xenograft tissues. Scale bar = 200 µm. **e** Co-localization of Amot-p130 (red) and β-catenin (green) in cell–cell contacts was indicated by immunofluorescence confocal microscopy. DAPI was used for nuclear staining (blue). **f** The interaction between Amot-p130 and β-catenin was evaluated in MCF7 cells by co-immunoprecipitation assay. IgG was used as the negative control. ***P < 0.001. DAPI, 4′,6-diamidino-2-phenylin

To elucidate the role of Amot-p130 in the WNT/β-catenin pathway, the protein levels of β-catenin were assessed by western blotting. Both cytoplasmic and nuclear β-catenin levels were increased by Amot-p130 knockdown in MCF7 cells (Fig. 5a). TOP/FOP luciferase reporter assays confirmed the higher level of β-catenin-driven transcription in MCF7KD than in MCF7 cells (Fig. 5b). Opposite results were observed in MM231OE cells. The WNT pathway controls the transcription of many important genes, including *Cyclin D1, TCF4, LEF, Met*, and *C-Myc*. The protein levels of these downstream targets were upregulated in MCF7KD cells and downregulated in MM231OE cells (Fig. 5c). IHC analysis showed that β-catenin levels were upregulated in MCF7KD-derived xenografts and downregulated in MM231OE xenografts (Fig. 5d). These results indicate that Amot-p130 inhibits the WNT pathway. Immunofluorescence staining showed the co-localization of Amot-p130 with β-catenin at cell–cell junctions in MCF7 control and MM231OE cells (Fig. 5e). The close relationship between Amot-p130 and β-catenin being revealed in the present study, we investigated whether Amot-p130 directly interacts with β-catenin. Co-immunoprecipitation assays showed that the anti-Amot-p130 antibody did not pull down β-catenin (Fig. 5f), suggesting that Amot-p130 inhibits WNT pathway activity without directly interacting with β-catenin.

### Amot-p130 regulates β-catenin stability by competing with Axin for binding to TNKS

**Fig. 6:**
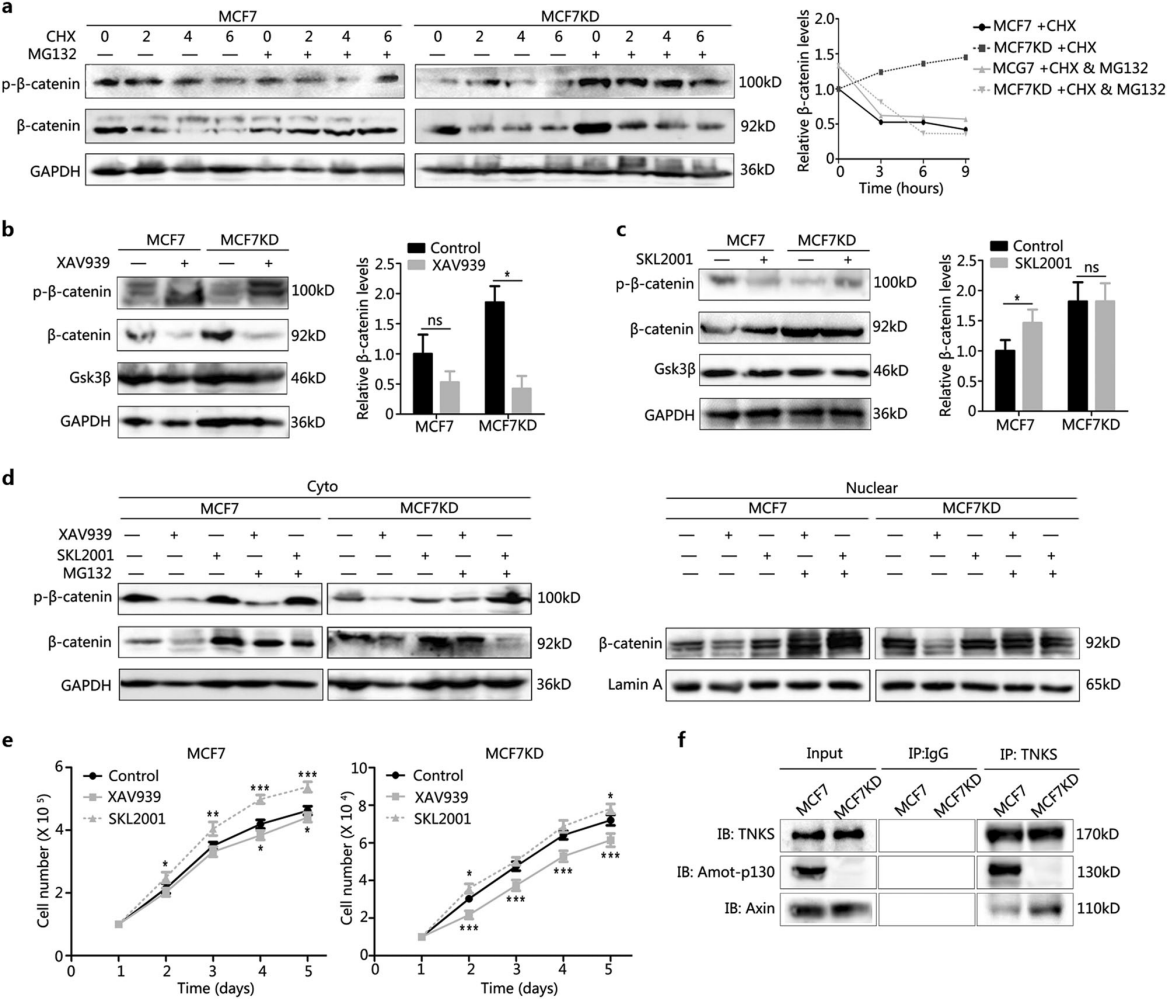
Amot-p130 regulates β-catenin stability by competing with Axin for binding to TNKS. **a** β-Catenin levels in cells treated with CHX (200 µg/ml) for the indicated time, with or without MG132 (20 µM) for 2 h, were determined using western blotting (left). GAPDH was used as the loading control. The curve showed the relative trend of β-catenin changes (right). **b** β-Catenin levels in cells treated with XAV939 10 µg/ml for 24 h were determined using western blotting (left). The quantitation of β-catenin was expressed as the mean ± SD of three independent experiments (right). **c** β-catenin levels in cells treated with SKL2001 30 µM for 24 h were determined using western blotting (left). The quantitation of β-catenin was expressed as the mean ± SD of three independent experiments (right). **d** Protein levels of the total, cytoplasmic, and nuclear β-catenin in cells treated with XAV939 or SKL2001, alone or in combination with MG132, were determeined using western blotting. GAPDH was used as the loading control for the total and cytoplasmic protein. Lamin A was used as the loading control for nuclear protein. **e** MCF7 cell proliferation under XAV939 or SKL2001 treatment was determined by MTT assay. **f** Relative quantitation of Amot-p130 and Axin pulled down by TNKS in co-immunoprecipitation assay. IgG pull-down was used as the negative control. *P < 0.05, **P < 0.01, ***P < 0.001; ns, no significance. MTT, 3-(4,5-dimethylthiazol-2-yl)-2,5-diphenyltetrazolium bromide; TNKS, tankyrase

Next, we explored the mechanism by which Amot-p130 affects β-catenin. Firstly, cells were treated with cycloheximide (CHX) to inhibit protein synthesis, alone or in combination with MG132. MG132 was added to prevent the ubiquitination-mediated degradation of β-catenin. The results showed that β-catenin protein levels decreased with time after a single CHX treatment in all the cell lines. The addition of MG132 to CHX-treated cells prolonged the half-life of β-catenin in MCF7 and MM231OE groups, but not in MCF7KD and MM231 groups (Fig. 6a and Supplementary Fig. S1a). This suggested that Amot-p130 was involved in the degradation of β-catenin. Next, the cells were treated with XAV939 to block the acetylation of Axin by TNKS and then induce the phosphorylation of β-catenin by the Axin-containing destruction complex. β-catenin levels only decreased significantly in MCF7KD and MM231 cells (Fig. 6b and Supplementary Fig. S1b). Lastly, the cells were treated with SKL2001, a β-catenin stabilizer, which resulted in β-catenin levels increasing significantly only in MCF7 and MM231OE cells (Fig. 6c and Supplementary Fig. S1c). Specifically, XAV939 treatment reduced the levels of nuclear β-catenin in MCF7KD and MM231 cells, whereas SKL2001 upregulated nuclear β-catenin in MCF7 and MM231OE cells (Fig. 6d and Supplementary Fig. S1d). The growth rates of MCF7 and MM231OE cells were slower in response to XAV939, whereas MCF7KD and MM231 cells grew faster upon SKL2001 treatment (Fig. 6e and Supplementary Fig. S1e).

TNKS plays an important role in the WNT pathway by directly binding to Axin. Recently, TNKS was also found to bind to the N-terminal domain of Amot-p130^27,28^. Hence, we speculated a competitive relationship between Amot-p130 and Axin in binding to TNKS. Our co-immunoprecipitation assays showed that less Axin was pulled down by the TNKS antibody in MCF7 and MM231OE cells (Amot-p130 positive cells) than in MCF7KD and MM231 cells (Amot-p130 negative cells) (Fig. 6f and Supplementary Fig. S1f). In summary, a higher proportion of TNKS bound to Amot-p130 than to Axin in Amot-p130 positive cells. Collectively, the data suggested that Amot-p130 regulates β-catenin stability by competing with Axin for binding to TNKS.

## Discussion

**Fig. 7:**
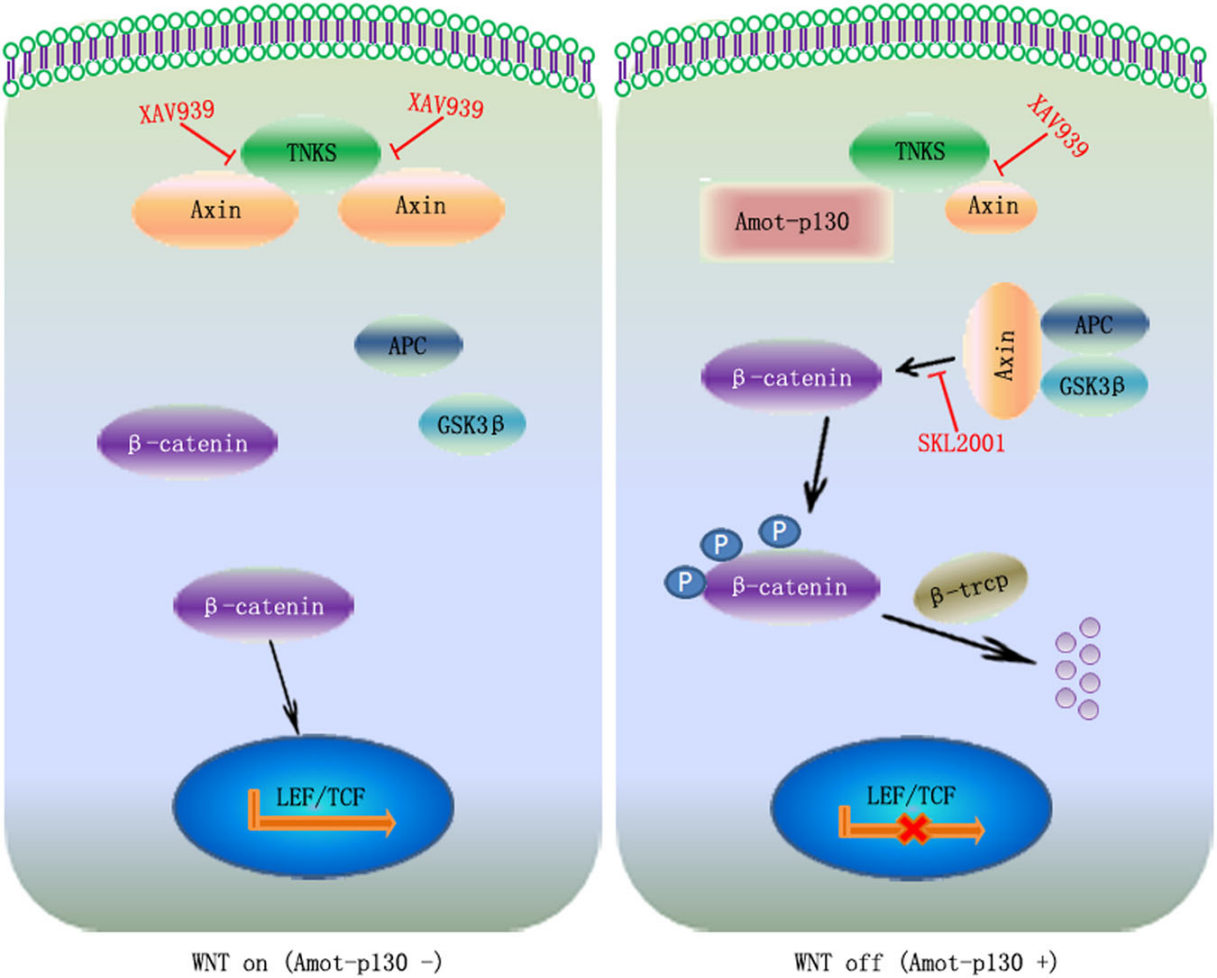
Model showing the regulation of the WNT pathway by Amot-p130. Axin/TNKS binding contributes to the nuclear translocation of β-catenin and the further activation of the WNT pathway in Amot-p130 negative cells (left). Amot-p130/TNKS binding allows Axin to form β-catenin-destruction complexes, leading to the phosphorylation and ubiquitination-mediated degradation of β-catenin, thereby inactivating WNT signaling in Amot-p130 positive cells (right). TNKS, tankyrase

The present study established the tumor suppressive role of Amot-p130 in BCa, in contrast with the canonical oncogenicity of Amot-p80 or total Amot. The results indicated that Amot-p130 inhibits WNT/β-catenin signaling by competing with Axin for binding to TNKS. As summarized in Fig. 7, Axin/TNKS binding induces the nuclear translocation of β-catenin and the activation of the WNT pathway (left panel). Amot-p130/TNKS binding allowed Axin to form β-catenin-destruction complexes, thereby inactivating WNT/β-catenin signaling (right panel) and inhibiting the CSC potential in BCa.

Unlike Amot-p130, the Amot-p80 isoform is oncogenic in BCa, as is total Amot^8,29^. Total Amot was expressed at higher levels in BCa tissues than in matched normal tissues, and BCa patients with high Amot expression were associated with a poorly differentiated disease, multiple metastases, and poor survival^10^. Additionally, in vitro studies show that total Amot promotes the proliferation and invasion of BCa cells^3^. The opposite roles of Amot isoforms in BCa are consistent with their roles in the regulation of endothelial cell migration^15^, as Amot-p130 stabilizes migration whereas Amot-p80 promotes migration. Migration is repressed in endothelial cells expressing only Amot-p130, whereas a relatively small amount of Amot-p80 compared with Amot-p130 is sufficient to induce a migratory phenotype^20^. Amot-p80 and Amot-p130 expression undergoes dynamic changes, and a balance between the expression of the two isoforms controls BCa phenotype. Amot-p80 expression is responsible for the advantageous oncogenic role of total Amot in BCa cell lines. We found that the ratio of Amot-p130 to Amot-p80 expression was high in luminal BCa cells, whereas it was low in triple-negative BCa cells. The tumor suppressive role of Amot-p130 was demonstrated in several cancers^4,5^. Clinically, low Amot-p130 expression is an independent indicator of poor survival in lung cancer patients^30^.

Amot-p130 is a key regulator of embryonic development and is involved in regulating visceral endoderm movement during mouse embryonic stem cell differentiation^31,32^. Amot-p130 loss leads to the differentiation of the inner cell mass^33^. Amot is one of the three genes showing expression alterations at the mRNA and protein levels in mesenchymal stem cells in the dermis of patients with psoriasis compared with healthy controls^34^.

However, little attention has been paid to the function of Amot-p130 in CSCs. Our work began by investigating the involvement of Amot-p130 in cell growth and EMT in BCa cells. We showed that the differentially expressed genes induced by endogenous Amot-p130 knockdown were mostly associated with breast CSCs, such as *ID4* and *CTGF*^35,36^. We further demonstrated that Amot-p130 inhibits breast CSC potential, including the regulation of CSC-enriched populations, drug resistance, the expression of stemness markers, tumor-sphere formation, and xenograft outgrowth.

The opposite functions of Amot-p130 and Amot-p80 are mostly mediated by the N-terminal domain. This extra domain is involved in several protein interactions, such as the classical interaction between the PPXY and the WW domains of YAP; therefore, Amot-p130 is an important effector in the HIPPO pathway^37^. In the present study, the WNT signaling pathway was ranked highest among the breast carcinogenesis-related pathways affected by Amot-p130 knockdown. Amot-p130 downregulated cytosolic and nuclear β-catenin, suppressed β-catenin-driven transcription, and decreased the protein levels of WNT downstream partners. The WNT pathway is crucial for regulating the self-renewal, migration, and tumorigenicity of breast CSCs^38,39^. Growing evidence indicates that Motin family members play important roles in the WNT pathway. AmotL2 reduces cytosolic and nuclear β-catenin levels by redirecting β-catenin into recycling endosomes, decreasing the tissue size during zebrafish development^24,40^. Mechanistically, the N-terminal glutamine-rich domain is essential for the interaction between AmotL2 and β-catenin. Moreover, the exogenous expression of Motin family members inhibits β-catenin-driven transcription in HEK293T cells^24^.

Amot-p130 is a classical component of cell-junction complexes, and β-catenin plays a central role in adherens junctions^41^. In the present study, Amot-p130 and β-catenin co-localized at the cell–cell junction, suggesting that the two molecules function together in junction formation. Given the similarity in structure between Amot-p130 and AmotL2, we hypothesized that Amot-p130 retains β-catenin at the cell–cell junction, leading to a further inhibition of WNT signaling. Consistent with the role of Amot-p130, the formation of cell junctions negatively affects cell growth, migration, and invasion^42^. In this case, the level of total β-catenin would decrease upon Amot-p130 silencing, because most of the free β-catenin released from the junction complex would be degraded in the cytoplasm. This is not the case, however. No direct interaction between Amot-p130 and β-catenin was detected. Instead, Amot-p130 may indirectly regulate β-catenin stability, as suggested by the present results showing the effect of Amot-p130 on (1) the protein levels of β-catenin and p-β-catenin and (2) the reversal of the decreasing trend of β-catenin levels induced by a co-treatment with MG132 and CHX.

TNKS regulates the degradation of Axin through binding and acetylation^43,44^. The specific PRPPVPGEE sequence in the N-terminal region of Axin and the ankyrin repeat domain of TNKS are required and sufficient for their physical interaction^45^. Similarly, Amot-p130 physically interacts with TNKS through the highly conserved sequence RQEPQGQE at its N terminus^27^. Compared with Amot-p130 negative cells, Amot-p130 positive cells showed (1) equal levels of the TNKS protein, (2) lower levels of Axin/TNKS binding, (3) decreased β-catenin degradation upon XAV939 treatment, and (4) decreased β-catenin phosphorylation upon SKL2001 treatment, leading to the nuclear translocation of β-catenin. We demonstrated that Amot-p130 competes with Axin for binding to TNKS in BCa. Hence, β-catenin-driven transcription and cell growth were significantly affected by SKL2001 treatment in Amot-p130 positive cells and by XAV939 in Amot-p130-negative cells. XAV939 treatment inhibits clone formation in MCF10A cells, but not in HEK293T cells^46^. To some extent, these results support our hypothesis, because HEK293T has high levels of Amot-p130.

Future studies need to be focussed on the effect of WNT inhibitor and/or agonist treatment on CSC potential. Whether Amo-p130 expression in BCa tissues is associated with clinicopathological features, metastasis, and prognosis needs to be evaluated. Future bioinformatics analyses should consider the different Amot isoform types.

In conclusion, we showed that Amot-p130 functions as a tumor suppressor in BCa, targeting CSC potential and leading to the inhibition of cell growth and EMT. Mechanistically, Amot-p130 inhibited the WNT/β-catenin pathway, modulating β-catenin stability by competing with Axin for binding to TNKS. The present study provides a new mechanistic insight into the regulation of Axin protein homeostasis and suggests new avenues for WNT pathway-targeted therapies in BCa.

## Methods

### Cell culture and reagents

MCF7 and MM231 cells were cultured in Dulbecco's modified Eagle's medium (DMEM) with 10% fetal bovine serum (HyClone) and maintained in a humidified incubator at 5% CO_2_ and 37 °C. Lentivirus targeting human Amot-p130(NM_001113490.1) were constructed by GeneChem (sequences, Table 1). Cells were infected using polybrene (Santa Cruz) and treated with puromycin for 3 weeks to generate stable Amot-p130-knockdown (MCF7KD) or Amot-p130-overexpression (MM231OE) cells. The antibody against Amot was synthesized by Genemed Synthesis Inc. The specific antibody against Amot-p130 was purchased from Santa Cruz BioTechnology (sc-166924). Antibodies against β-catenin (#8084P), p-β-catenin (#9561T), YAP (#14074S), TAZ (#4883), vimentin (#5741P), E-cadherin (#3195P), OCT-4 (#2750), Nanog (#4903P), SOX2 (#3579P), C-myc (#5605), Cyclin D (#2978), LEF1 (#2230P), TCF4 (#2565), MET (#8198), Gsk3β (#12456T), and β-trcp (#4394S) were from Cell Signaling Technology. HRP-labeled GAPDH (HRP-60004) and antibody against lamin A (10298-1-AP) were from Proteintech Group. XAV939 and SKL2001 were from Selleck Chemicals.

**Table 1:** Sequences of shRNA and primers used

Name	Sequence
Amot-p130 shRNA #1	CATACACCAGCAAGCCACAGGGAAT
Amot-p130 shRNA #2	CAAGAATCCCACAAGTTCCAGTGAA
Amot-p130-F	AGCCTGCTTGCCATACACC
Amot-p130-R	CTTCTTCATAGGTCGGGAGTTCTT
GAPDH-F	CTCCTCCACCTTTGACGCTG
GAPDH-R	TCCTCTTGTGCTCTTGCTGG
ETS1-F	TGTATTTTGCATCCCTGGTT
ETS1-R	AACGACATCGATTCAGGACT
TCF4-F	GAGGCAGCCATTCTCTTCTG
TCF4-R	CAGGTTCTCATCACCCTCGT
ID1-F	GGAATCCGAAGTTGGAACC
ID1-R	CGCTTCAGCGACACAAGAT
ID4-F	CCACCATCCCGCCCAACAAG
ID4-R	CTCCCTCTCTAGTGCTCCTG
CTNND1-F	ACCTGAGGAGCCCCAACAA
CTNND1-R	TCTGCTCCTGGCAGGCC
ANXA6-F	CTGGACATAATCACCTCACG
ANXA6-R	TTGGCATCACAATAGGCAGG

### Cell count assay

Cells were seeded in 24-well plates at a density of 4 × 10^4^ cells/well for MCF7 group and 2 × 10^4^ cells/well for MM231 group. After culturing for the indicated time points, cells were washed with phosphate-buffered saline (PBS), detached with 0.25% trypsin for 10 min at 37 °C, and resuspended with an equal volume of the growth medium. Viable cells were counted with a hemocytometer under an inverted microscope system. The experiment was performed in triplicate.

### Wound-healing assay

Cells were seeded in six-well plates at a density of 1 × 10^6^ cells/well for MCF7 group and 5 × 10^5^ cells/well for MM231 group, and cultured in complete medium for 24 h. Following the removal of the culture medium, a monolayer of the sub-confluent cells was scratched with a 200 µl pipette tip to create a wound area. The wounded monolayer was washed with PBS twice and cultured for 24 h in 2% fetal bovine serum (FBS) medium for MCF7 group or FBS-free medium for MM231 group. Cell migration into the wound area was monitored by inverted microscopy and photographed at the indicated time points. The experiment was performed in triplicate.

### Cell cycle and apoptosis assay

For the cell cycle assay, 1 × 10^6^ cells were fixed overnight in 70% ethanol and stained using 10 μg/ml propidium iodide (Sigma-Aldrich) and 50 μg/ml RNase A (Sigma-Aldrich). For apoptosis assay, 1 × 10^5^ cells were collected and stained with 7AAD and Annexin V (#559763, Apoptosis Detection Kit; BD Bioscience). Both data were analyzed by flow cytometry (BD Biosciences).

### Migration and invasion assays

Migration and invasion assays were performed using the BioCoat cell migration chamber (Corning), which consists of a 24-well companion plate with cell culture inserts containing a filter with 8 µm-diameter pores. The transwell for the invasion assay was coated with matrigel (1:4 dilution with FBS-free DMEM; Corning). The cells were trypsinized, suspended with FBS-free DMEM, and seeded at 2 × 10^5^/ml for the migration assay and at 2 × 10^5^/ml for the invasion assay. Hundred microlliters of the cell suspension was added to the upper well, and 600 µl of DMEM containing 10% FBS was added to the lower well. The plates were placed in 5% CO_2_ at 37 °C and observed every 4 h. When the appearance of cells was observed in the lower well, the cells in the upper wells were gently removed by scrubbing, fixed in 95% ethanol for 15 min, and stained with 0.4% crystal violet for 30 min. The migrated or invaded cells were subsequently photographed with a microscope. The experiment was performed in triplicate.

### Microarray

Total RNA was purified from cells using Trizol and examined using Thermo NanoDrop 2000. After purification and amplification (GeneChip), the RNA was hybridized onto Human Gene 1.0 ST arrays (Affymetrix). Raw data were normalized using Robust Multichip Array and log2 transformed using BRB-ArrayTools v4.3.0. Differentially expressed genes between MCF7 and MCF7KD cells were defined by *P* < 0.05 and by a fold change > 1.5 (Supplementary Table S1). Gene Ontology and Ingenuity Pathway Analysis were performed to identify enriched functionally associated pathways.

### Tumor-sphere formation

Cells were plated at a density of 10000 cells/well for MCF7 and 5000 cells/well for MM231 in ultra-low attachment six-well plates and cultured in DMEM/F12 medium containing 20 μg/ml epidermal growth factor and 2% B27. Cells were maintained in 5% CO_2_ at 37 °C, and the medium was replaced every 3 days. The total number of spheres greater than 80 μm in diameter was counted at 14 days under an inverted microscope.

### Luciferase reporter assay

Cells were plated at a concentration of 5000 cells/well in 96-well plates. After overnight serum starvation, the cells were co-transfected with 0.2 µg TOPflash or FOPflash plasmids (Addgene) and 0.1 µg Renilla TK-luciferase vector (Promega) as a control using Lipofectamine 2000 (Invitrogen). Luciferase activity was measured by the Dual-Luciferase Reporter Assay (Promega) using a Glomax 96 Microplate Luminometer 48 h later. The firefly luciferase activity was normalized to the internal Renilla luciferase actvity, and the β-catenin-driven transcription was measured as TOP/FOP ratio.

### Surface staining by flow cytometry

Trypsinized cells were washed twice with PBS, blocked with 2% bovine serum albumin, and stained with anti-CD44-APC conjugate (Biolegend) and anti-CD24-PE conjugate (Biolegend) in dark at 4 °C for 30 min. After washing twice, the cells were analyzed by flow cytometry (Becton Dickinson) using Cell Quest Pro software. The ALDEFLUOR kit (Stem Cell) was used to detect intracellular aldehyde dehydrogenase (ALDH) enzyme activity. One million cells were suspended in ALDEFLUOR assay buffer containing the ALDH substrate and incubated for 60 min at 37 °C. As a negative control, an aliquot of each cell sample was treated with diethylaminobenzaldehyde, a specific ALDH inhibitor.

### MTT assay

Cells were seeded in 96-well plates at a density of 5 × 10^3^ cells/well for MCF7 group and 2 × 10^3^ cells/well for MM231 group. Twenty microliters of 5 mg/ml 3-(4,5-dimethylthiazol-2-yl)-2,5-diphenyltetrazolium bromide (MTT) solution (Sigma) was added to each well and incubated for 4 h. After the removal of the supernatant, 150 μl of dimethyl sulfoxide was added to each well. The plates were kept in the dark and slightly shaken for 30 min at room temperature. The OD values of each well were measured by a microplate reader at a wavelength of 490 nm. The experiment was performed in triplicate.

### Xenograft and limited-dilution assays

NOD-SCID female mice aged 4 weeks were bred at a specific pathogen-free animal facility. For xenograft assays, 5 × 10^6^ cells diluted in 100 μl PBS were injected into the mammary fat pad of mice on both the flanks. For limited-dilution assays, cells were injected at concentrations of 5 × 10^3^, 5 × 10^4^, 5 × 10^5^, and 2 × 10^6^ cells into the fat pads (*n* = 6). Mice were monitored for tumor development twice a week using a caliper. All the mice were euthanized at the end of the 6th week. Xenograft tumors were measured, weighed, and analyzed by IHC as previously described^37^. All animal procedures complied with the guidelines of the Institutional Animal Use and Care Committee and were approved by the Animal Ethics Committee of Xi’an Jiaotong University.

### Other assays

Real-time quantitative PCR (primers, Table 1) was performed as previously described^10^. Assays including western blotting, immunofluorescence, plate clone formation, and immunoprecipitation were also done as previously described^36^.

### Statistical analysis

Statistical analyses were performed using GraphPad Prism software (version 5.0). Data were analyzed by paired Student's *t* test and presented as mean ± SEM. *P* < 0.05 at two sides was considered statistically significant. The asterisks *, **, and *** stand for *P* less than 0.05, 0.01, and 0.001, respectively. Statistical calculations were derived from at least three independent replicates.

## Supplementary information

Table S1

Figure S1

supplemental figure legends

## Data Availability

All the data generated or analyzed during this study are included in this published article (and its supplementary information files).
