# Supplementary figures and images for: Angiomotin-p130 inhibits β-catenin stability by competing with Axin for binding to tankyrase in breast cancer
Source: Cell Death Dis. 2019 Feb 21;10(3):179. doi: 10.1038/s41419-019-1427-2 (PMC6385204; doi:10.1038/s41419-019-1427-2)

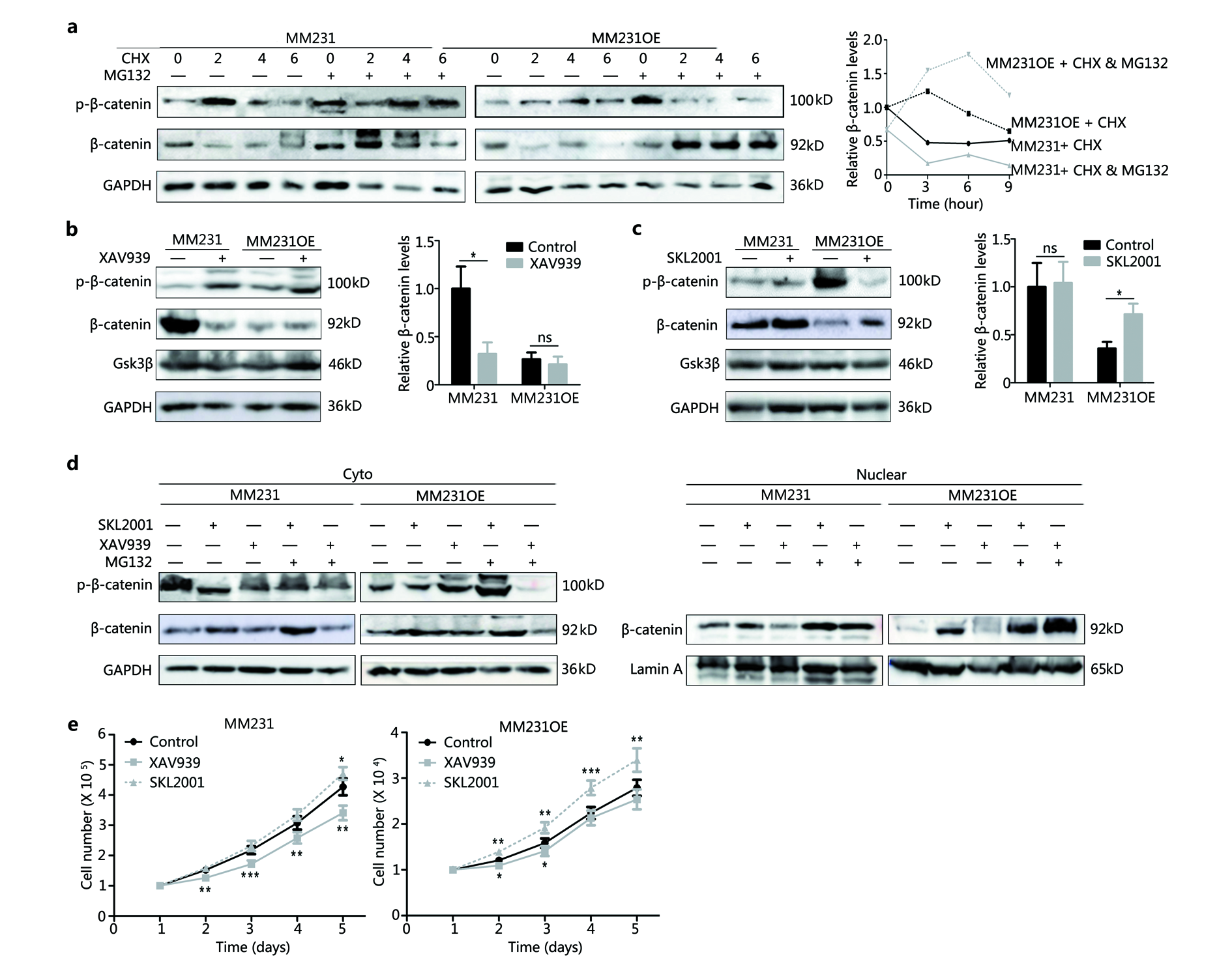

Supplement: Supplementary file 2 — Figure S1 [file 41419_2019_1427_MOESM2_ESM.tif]
