# Supplementary material for: Angiomotin-p130 inhibits β-catenin stability by competing with Axin for binding to tankyrase in breast cancer
Source: Cell Death Dis. 2019 Feb 21;10(3):179. doi: 10.1038/s41419-019-1427-2 (PMC6385204; doi:10.1038/s41419-019-1427-2)
Supplement: Supplementary file 3 — supplemental figure legends [file 41419_2019_1427_MOESM3_ESM.docx]

**Additional file 2.**

Figure S1. Amot-p130 regulates β-catenin stability by competing with Axin for binding to TNKS in MM231 cells.

(a) β-catenin levels in cells treated with CHX (200 µg/ml) for the indicated time in combination with or without MG132 (20 µM) for 2 hours were determeined using western blotting (left). GAPDH was used as loading control. The curve showed the relative trend of β-catenin changes (right). (b) β-catenin levels in cells treated with XAV939 (10 µg/ml) for 24 hour were determeined using western blotting (left). The quantitation of β-catenin was expressed as the mean ± SD of three independent experiments (right). (c) β-catenin levels in cells treated with SKL2001 (30 µM) for 24 hours were determeined using western blotting (left). The quantitation of β-catenin was expressed as the mean ± SD of three independent experiments (right). (d) Protein levels of total, cytoplasmic, and nuclear β-catenin in cells treated with XAV939 or SKL2001, alone or in combination with MG132 were determeined using western blotting. GAPDH was used as loading control for total and cytoplasmic protein. Lamin A was used as loading control for nuclear protein. (e) MM231 cell proliferation under XAV939 or SKL2001 treatment was determined by the MTT assay. * *P*<0.05, ** *P*<0.01, *** *P*<0.001; ns, no significance.
